# Pertussis in the Tshwane District, Gauteng province, South Africa: A cross-sectional study

**DOI:** 10.4102/jphia.v16i1.1393

**Published:** 2025-10-29

**Authors:** Xolelwa Ntsham, Tladi D. Ledibane

**Affiliations:** 1 Department of Public Health Medicine, School of Medicine, Sefako Makgatho Health Sciences University, Pretoria, South Africa

**Keywords:** pertussis, whooping cough, vaccines, infants, public health, surveillance data, cross-sectional study, risk factors, seasonal variation, vaccination strategies, disease control, Tshwane District

## Abstract

**Background:**

Pertussis remains a public health concern worldwide, particularly in infants and young children. Despite effective vaccines, challenges persist in addressing pertussis because of barriers such as limited healthcare access, inadequate vaccination coverage and weak surveillance systems, especially in low- and middle-income countries.

**Aim:**

To describe the demographic, geographic and seasonal distribution of pertussis cases reported in Tshwane District, Gauteng province, South Africa, from 2015 to 2019.

**Setting:**

The study was conducted in Gauteng’s Tshwane District, which has seven sub-districts with diverse demographics ranging from highly urbanised to peri-urban and rural areas.

**Methods:**

A retrospective cross-sectional analysis of surveillance data was conducted for all pertussis cases notified in Tshwane between 1 January 2015 and 31 December 2019. Data were obtained from the district Pertussis Monitoring Database and validated against the District Health Information System. Descriptive statistics, chi-square tests and time-series analyses were applied.

**Results:**

A total of 272 cases were reported; 46.7% occurred in infants under 1 year, with those younger than 6 months comprising 39.7%. One-third were hospitalised, mostly infants. Most notifications originated from private facilities (73.5%), and spring–summer peaks were observed. Three infant deaths were recorded.

**Conclusion:**

Pertussis contributes substantially to the disease burden in Tshwane, particularly in early infancy. Strengthened immunisation programme, improved surveillance and equitable diagnostic access are essential to reduce morbidity and mortality.

**Contribution:**

This study provides district-level analysis of pertussis in Tshwane, highlighting inequities in vaccination reporting and diagnostic access and informing strategies to strengthen pertussis immunisation and surveillance.

## Introduction

Pertussis, also known as whooping cough, is a highly contagious respiratory disease caused by the bacterium *Bordetella pertussis*. It is characterised by severe coughing fits, often accompanied by a characteristic ‘whooping’ sound during inhalation. Pertussis can affect individuals of all ages, but it is particularly severe and even life-threatening in infants and young children.^1^

Despite the availability of effective vaccines, pertussis continues to be a significant public health concern worldwide. According to estimates from the Global Vaccine Action Plan, there were approximately 151 000 pertussis-related deaths globally in 2019,^2^ with the majority of fatalities occurring in infants under the age of five.^1^ This highlights the vulnerability of young children to the disease and the urgent need for effective prevention and control strategies.^3^

One of the primary challenges in addressing pertussis is the persistence of the disease in low- and middle-income countries (LMICs). These regions often face barriers such as limited access to healthcare services, inadequate vaccination coverage and weak surveillance systems.^4,5,6,7^ For example, only two African countries reached the 90% vaccine coverage target, with many other countries having high dropout rates ranging from 50% to 30%.^4^ As a result, the true burden of pertussis in LMICs is likely underestimated, and the disease remains a significant cause of morbidity and mortality among infants and young children.

Another challenge is the waning effectiveness of pertussis vaccines over time.^8^ While vaccines have significantly reduced the incidence of pertussis in many countries, there have been reports of outbreaks and increasing cases among adolescents and adults.^9^ This suggests that immunity acquired through vaccination or natural infection may decrease over time, leaving individuals susceptible to infection and contributing to the ongoing transmission of the disease. In addition, maternal antibodies offer temporary protection to unvaccinated infants,^10^ but their levels decline quickly during the early months of life, making infants susceptible to infections and severe health outcomes.^11^

Respiratory infections are usually seasonal, but there is ongoing debate regarding whether pertussis infections exhibit a seasonal pattern.^12,13^ Some studies indicate that there are seasonal fluctuations in pertussis incidence, with higher rates during spring and summer.^13^ However, other studies suggest that pertussis infections are more prevalent in winter or provide inconclusive results.^14,15^ The reasons for these seasonal fluctuations are not fully understood, but may be influenced by factors such as changes in respiratory infections, environmental conditions and human behaviour.

This study aims to describe the trends in pertussis infections in the Tshwane District from 01 January 2015 to 31 December 2019. The objectives were to describe the demographic and geographic profiles of pertussis infections and the seasonal differences in pertussis infections in the Tshwane District.

## Research methods and design

### Study design

This study employed a retrospective review of cross-sectional data collected about pertussis in the period from 01 January 2015 to 31 December 2019.

### Study setting

The Tshwane District, located in Gauteng province, South Africa, served as the study setting. This metropolitan area borders the provinces of Limpopo, Mpumalanga and North West. The district consists of seven subdistricts (Regions 1–7), each characterised by diverse demographics, ranging from the highly urbanised central regions to rural and peri-urban areas. Tshwane District has an estimated population of 3.5 million, with the largest share within the young working-age category (25–44 years, 36.5%) and the second largest being children aged 0–14 years (24.5%).

### Case definition and eligibility criteria

Pertussis cases were identified in accordance with the South African National Institute for Communicable Diseases (NICD) case definition: any individual presenting with a cough lasting seven or more days accompanied by paroxysms, inspiratory whoop or post-tussive vomiting, or any person with a compatible clinical presentation and laboratory confirmation *via* polymerase chain reaction (PCR) or culture.^16^ Both laboratory-confirmed and clinically diagnosed cases were eligible.

### Surveillance programme description

Pertussis is classified as a Category 1 notifiable medical condition (NMC) in South Africa, requiring notification within 24 h of clinical suspicion or laboratory confirmation.^16^ Pertussis surveillance in Tshwane is facility-based, where suspected pertussis cases are identified and notified by clinicians or Infection Control Practitioners (ICPs) within 24 h of clinical suspicion or laboratory confirmation. Notifications are transmitted to the district surveillance office *via* various channels, including email, paper-based forms, short message services (SMS), WhatsApp and the electronic NMC application system.^17^

### Data collection and management

The data were retrieved retrospectively from two surveillance sources. The Pertussis Monitoring Database, maintained by the Tshwane District surveillance team, is a line-list database that captures individual-level information only for notified pertussis cases. For each case, the data entered include demographic details such as age and sex, geographic identifiers, vaccination status, clinical outcomes, hospital type (public or private), and specimen collection and laboratory confirmation results. The vaccination status was categorised as up-to-date, not vaccinated, unknown or not applicable for cases not yet eligible for vaccination. The second source was the District Health Information System (DHIS2, version 2.30), which provides aggregate monthly counts of NMC, including pertussis, as reported by health facilities. The DHIS2 data were used to cross-check completeness of the line-list dataset and to validate monthly case counts for trend analysis.

All extracted data were captured into a Microsoft Excel spreadsheet for cleaning, where inconsistencies, inaccuracies and missing values were identified and corrected. Following this step, the cleaned dataset was exported into Stata version 17.0 (StataCorp LLC, Texas, United States) for statistical analysis.

### Data analysis

All notified pertussis cases, including both laboratory-confirmed and clinically diagnosed cases, were included in the analysis. Descriptive statistics were used to summarise both demographic and clinical features. Categorical variables were presented as frequencies and percentages, while continuous variables were described using means, medians, ranges and standard deviations. Associations between categorical variables were examined using Chi-square tests of association, with statistical significance set at *p* < 0.05.

Temporal trends were evaluated by plotting monthly case counts and applying a 3-month centred moving average to smooth short-term fluctuations. Seasonal variation was assessed by grouping cases into calendar seasons and comparing case frequencies, while spatial heterogeneity was examined descriptively by analysing case counts and proportions across subdistricts.

### Ethical considerations

The authors obtained permission to conduct the study from the Gauteng Department of Health, and ethical clearance for the study was obtained from the Sefako Makgatho Health Science University Research Ethics Committee (SMUREC) (No. SMUREC/M/58/2021). Consent was not needed from the participants, as anonymised aggregated data were used. Sensitive information obtained during the study was treated as confidential. The principles of safeguarding the dignity and safety of all research participants as stipulated in the Declaration of Helsinki were observed by ensuring the privacy of records.

## Results

A total of 272 pertussis cases were reported in Tshwane from 2015 to 2019. Infants under 1 year of age constituted nearly half of the cases (46.7%), with the < 6 months age group accounting for 39.7% of total cases. Gender distribution was nearly equal: 50.7% female and 49.3% male. Geographically, Subdistrict 3 reported the highest number of cases (44.1%), followed by Subdistrict 6 (18.4%) and Subdistrict 4 (15.8%). Most reported cases originated from private health facilities (73.5%), and a notable seasonal variation was observed, with spring (36.4%) accounting for the highest proportion of cases.

Table 1 provides an overview of the demographic and geographic characteristics of reported pertussis cases, showing a preponderance of cases among infants under the age of 6 months, an evenly balanced gender a distribution and higher proportion of reported cases from the urban areas.

**TABLE 1:** Demographic and geographic characteristics by sex.

Characteristic	Category	Female		Male		Total		*P*
		*n*	%	*n*	%	*n*	%	
**Age category**	-	-	-	-	-	-	-	0.151
	< 6 months	53	38.4	55	41.0	108	39.7	-
	6 months to < 1 year	8	5.8	11	8.2	19	7.0	-
	1–4 years	26	18.8	23	17.2	49	18.0	-
	5–9 years	15	10.9	20	14.9	35	12.9	-
	10–19 years	10	7.2	14	10.4	24	8.8	-
	20+ years	26	18.8	11	8.2	37	13.6	-
**Subdistrict**	-	-	-	-	-	-	-	0.177
	1	10	7.2	10	7.5	20	7.4	-
	2	7	5.1	9	6.7	16	5.9	-
	3	62	44.9	58	43.3	120	44.1	-
	4	22	15.9	21	15.7	43	15.8	-
	5	14	10.1	5	3.7	19	7.0	-
	6	23	16.7	27	20.1	50	18.4	-
	7	0	0.0	4	3.0	4	1.5	-
**Region type**	-	-	-	-	-	-	-	0.078
	Urban	107	77.5	106	79.1	213	78.3	-
	Peri-urban	17	12.3	23	17.2	40	14.7	-
	Rural	14	10.1	5	3.7	19	7.0	-

Table 2 compares the distribution of demographic and geographic variables by laboratory confirmation status. Subdistrict 5 and rural regions had significantly higher proportions of laboratory-confirmed pertussis cases, whereas age category and sex were not statistically associated with laboratory confirmation.

**TABLE 2:** Demographic and geographic characteristics stratified by laboratory confirmation status.

Characteristic	Category	Lab-confirmed no		Lab-confirmed yes		Total		*P*
		*n*	%	*n*	%	*n*	%	
**Age category**	-	-	-	-	-	-	-	0.339
	1–4 years	41	20.4	8	11.3	49	18.0	-
	10–19 years	16	8.0	8	11.3	24	8.8	-
	20+ years	26	12.9	11	15.5	37	13.6	-
	5–9 years	27	13.4	8	11.3	35	12.9	-
	6 months to < 1 year	16	8.0	3	4.2	19	7.0	-
	< 6 months	75	37.3	33	46.5	108	39.7	-
**Subdistrict**	-	-	-	-	-	-	-	< 0.001
	1	17	8.5	3	4.2	20	7.4	-
	2	12	6.0	4	5.6	16	5.9	-
	3	88	43.8	32	45.1	120	44.1	-
	4	29	14.4	14	19.7	43	15.8	-
	5	1	0.5	18	25.4	19	7.0	-
	6	50	24.9	0	0.0	50	18.4	-
	7	4	2.0	0	0.0	4	1.5	-
**Region type**	-	-	-	-	-	-	-	< 0.001
	Peri-urban	33	16.4	7	9.9	40	14.7	-
	Rural	1	0.5	18	25.4	19	7.0	-
	Urban	167	83.1	46	64.8	213	78.3	-

Hospitalisation outcomes are summarised in Table 3. About a third of cases (33.1%, 90/272) required hospital admission. Infants younger than 6 months accounted for the largest proportion of hospitalised cases (42.2%), and the association between age group and hospitalisation was statistically significant (*p* = 0.012). Hospitalisation did not differ significantly by sex or laboratory confirmation status. However, significant disparities were observed by region type, with peri-urban areas showing the highest proportion of hospitalisations compared with urban and rural areas (*p* < 0.001). Vaccination status was poorly documented, but hospitalised cases were disproportionately recorded as ‘unknown’ compared with outpatients (*p* < 0.001). Mortality was low, with three deaths (1.1%) documented among outpatients only, all in infants younger than 12 months. Hospital category showed marked inequalities: most admissions occurred in private hospitals (87.8%), while only 12.2% were admitted to public hospitals (*p* < 0.001).

**TABLE 3:** Comparison of demographic, geographic, vaccination, mortality and hospital category by hospitalisation status, Tshwane District, 2015–2019 (*N* = 272).

Characteristic	Category	Hospitalised		Not hospitalised		Total		*P*
		*n*	%	*n*	%	*n*	%	
**Age group**	-	-	-	-	-	-	-	0.012
	< 6 months	38	42.2	70	38.5	108	39.7	-
	6–11 months	0	0.0	19	10.4	19	7.0	-
	1–4 years	22	24.4	27	14.8	49	18.0	-
	5–9 years	11	12.2	24	13.2	35	12.9	-
	10–19 years	5	5.6	19	10.4	24	8.8	-
	≥ 20 years	14	15.6	23	12.6	37	13.6	-
**Sex**	-	-	-	-	-	-	-	0.871
	Female	45	50.0	93	51.1	138	50.7	-
	Male	45	50.0	89	48.9	134	49.3	-
**Laboratory confirmation**	-	-	-	-	-	-	-	0.412
	Confirmed	18	20.0	46	25.3	64	23.5	-
	Not confirmed	72	80.0	136	74.7	208	76.5	-
**Region type**	-	-	-	-	-	-	-	< 0.001
	Urban	65	72.2	148	81.3	213	78.3	-
	Peri-urban	25	27.8	15	8.2	40	14.7	-
	Rural	0	0.0	19	10.4	19	7.0	-
**Vaccination status**	-	-	-	-	-	-	-	< 0.001
	Up-to-date	0	0.0	16	8.8	16	5.9	-
	Not vaccinated	0	0.0	6	3.3	6	2.2	-
	Unknown	90	100.0	93	51.1	183	67.3	-
	Not applicable†	0	0.0	1	0.5	1	0.4	-
**Mortality**	-	-	-	-	-	-	-	0.221
	Died	0	0.0	3	1.6	3	1.1	-
	Survived	90	100.0	179	98.4	269	98.9	-
**Hospital category**	-	-	-	-	-	-	-	< 0.001
	Public	11	12.2	61	33.5	72	26.5	-
	Private	79	87.8	121	66.5	200	73.5	-

† , Not applicable = one case with missing vaccination eligibility data.

**FIGURE 1:**
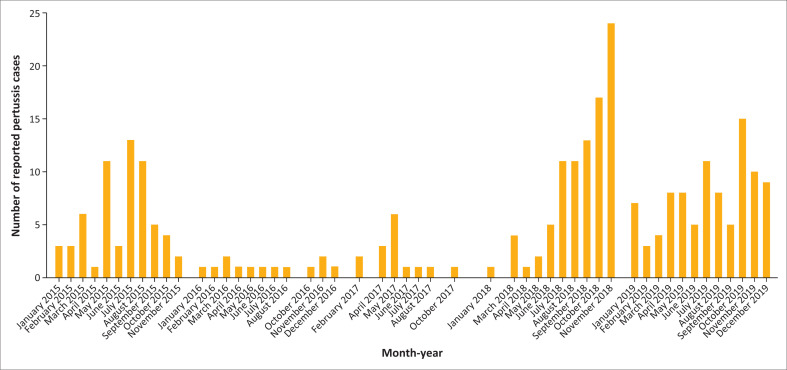
Temporal distribution of reported pertussis cases over the study period (2015–2019).

Figure 1 depicts the temporal distribution of reported pertussis cases throughout the duration of the study. Two distinct peaks were identified: the first occurring between June 2015 and November 2015, and a second, more pronounced peak, spanning September 2018 to March 2019. These peaks indicate episodic surges in the incidence of pertussis, in alignment with cyclical outbreak patterns. A reduction in case numbers was observed during 2016 and the early part of 2017.

**FIGURE 2:**
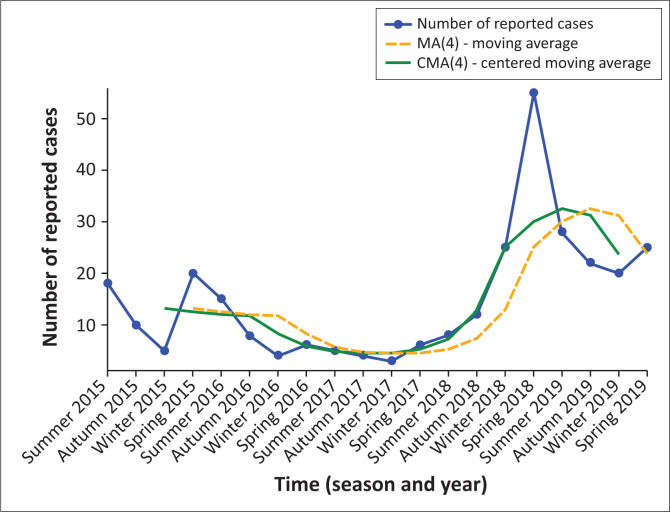
The seasonal trend in pertussis cases over time.

Figure 2 depicts the seasonal trend in pertussis cases over a temporal span, presenting quarterly aggregates by season and year. A 4-point moving average (MA4) and a centred moving average (CMA4) have been superimposed to attenuate short-term fluctuations and accentuate the underlying pattern. The figure demonstrates a resurgence of pertussis commencing in late 2017, with a pronounced peak in the summer of 2019. The sustained elevation into the winter and spring of 2019 indicates a prolonged outbreak period, underscoring seasonal clustering during spring and early summer.

## Discussion

This study provides a comprehensive epidemiological description of pertussis cases reported in Tshwane Health District, Gauteng province, South Africa, from 2015 to 2019. The findings of this study highlight demographic, geographic, seasonal and clinical heterogeneity, which bear important implications for pertussis control strategies in Tshwane. In addition, the findings did not identify any significant sex differences in pertussis occurrence or severity, indicating no evidence of gender disparity. This finding is consistent with global literature, which generally shows pertussis risk is driven more strongly by age, vaccination status and exposure dynamics rather than sex-based differences.^18,19^

Infants under 6 months of age constituted the largest proportion of pertussis cases, indicating their increased vulnerability prior to the completion of the primary immunisation series, a finding that aligns with global and South African studies.^18,19^ This finding demonstrates the need for maternal vaccination programmes to confer passive immunity to young infants.^10,11^

An important observation from this study is the marked geographic variation in pertussis case distribution and laboratory confirmation rates across district. Rural regions exhibited higher laboratory confirmation proportions, which may reflect differences in laboratory capacity or surveillance intensity.^20^ These disparities highlight the importance of ensuring equitable diagnostic service access and uniform surveillance practices across urban, peri-urban and rural settings.

Hospitalisation patterns provide further insight into disease severity and systemic inequities.

Infants younger than 6 months accounted for over 40% of hospitalisations, reinforcing their susceptibility to severe outcomes and the critical need for maternal vaccination during pregnancy and timely infant immunisation.^5,11^ The pronounced geographical disparity in hospitalisation rates, particularly evident in peri-urban subdistricts, suggests variations in healthcare access, socio-economic determinants and healthcare-seeking behaviours that must be addressed through targeted public health interventions and equitable resource allocation.^4,20^ Additionally, the significant disparity observed between private and public healthcare facility utilisation highlights systemic inequalities that require policy-level attention to ensure equitable access to quality healthcare across different socio-economic groups.^19,21^

Furthermore, nearly 90% of hospital admissions occurred in private facilities, compared with only 12% in public hospitals. This stark disparity indicates systemic inequities in diagnostic and inpatient care access, with potential implications for representativeness of surveillance data. Strengthening public sector diagnostic capacity and inpatient care is therefore essential to ensure both equity and accuracy in monitoring pertussis burden.

Seasonal trends revealed distinct peaks in pertussis cases during spring and summer, with a marked resurgence from September 2018 to March 2019. These temporal patterns are consistent with cyclic pertussis outbreaks observed in other settings.^22^ The multivariable analysis confirmed the independent association between seasonality and laboratory-confirmed pertussis, emphasising the need for heightened surveillance during peak transmission periods.

From a vaccination perspective, South Africa uses the acellular pertussis vaccine, which has fewer side effects than whole-cell vaccines. However, the acellular pertussis vaccine is associated with waning immunity.^8^ Furthermore, South Africa does not offer pertussis booster vaccines. The lack of an adolescent pertussis booster further contributes to susceptibility among older children and adolescents, potentially fuelling community transmission.^23^ Laboratory confirmation, while valuable, is not required for notification because pertussis is a Category 1 notifiable condition.^16^ Furthermore, all clinically suspected cases should be reported, regardless of whether a specimen is collected. This recommendation is particularly pertinent in the South African context, where routine PCR testing for pertussis is frequently unavailable in the public sector because of cost and laboratory capacity constraints. Consequently, surveillance data disproportionately reflect private sector testing, potentially biasing the understanding of pertussis epidemiology and underestimating the true burden in public healthcare settings.

The high proportion of cases with unknown vaccination status reveals gaps in health information system completeness, which may compromise vaccine coverage assessments and programme monitoring.^4,21^ Addressing this limitation requires strengthening routine data collection systems, potentially through digital health interventions and standardised reporting protocols.

The findings of this study also documented three deaths attributed to pertussis, all occurring among infants. This is consistent with hospital-based surveillance findings in South Africa, where infant pertussis mortality remains a concern.^19^ These deaths underline the importance of cocooning strategies, where household contacts and caregivers are vaccinated to protect vulnerable infants.^5,24^

### Limitations of the study

Although our study offered useful insights, it encountered limitations, such as potential underreporting and reporting biases, inherent in the surveillance data because of possible errors resulting from missing or incomplete records during data collection. Unexplored factors, such as variations in healthcare-seeking behaviour or biological characteristics, may have influenced the outcomes. Ultimately, the research was limited to the Tshwane Health District in Gauteng, so its findings may not be generalisable to other contexts.

## Conclusion

This study highlights the persistent burden of pertussis in the Tshwane Health District, particularly among infants younger than 6 months, who are at the highest risk for severe outcomes. The seasonal and geographic heterogeneity of reported cases underscores the need for intensified, context-specific surveillance and response strategies.

The high proportions of unknown vaccination status and limited laboratory confirmation rates among the reported cases indicate deficiencies in routine data quality and diagnostic coverage. Addressing these challenges requires investment in digital health information systems, laboratory capacity and standardised case investigation protocols.

Furthermore, to prevent pertussis-related morbidity and mortality in early infancy, maternal immunisation should be prioritised as part of the Expanded Programme on Immunisation (EPI), alongside improved outreach and health education in high-burden subdistricts. The findings also justify further research using sero-epidemiology to better understand disease dynamics and inform evidence-based policy.

A coordinated strategy that includes enhanced maternal vaccination, improved surveillance and equitable access to diagnostics is essential to reduce the burden of pertussis in Tshwane.
